# Optimizing Use of Real‐World Data in Regulatory Decision Making for Medical Devices Used in Surgical Procedures

**DOI:** 10.1002/pds.70407

**Published:** 2026-06-07

**Authors:** Guy Cafri, Florence Bourgeois, Paul Coplan

**Affiliations:** ^1^ Epidemiology and Real‐World Data Sciences Johnson & Johnson MedTech, Office of the Chief Medical Officer New Brunswick New Jersey USA; ^2^ Department of Pediatrics Harvard Medical School Boston Massachusetts USA; ^3^ Harvard‐MIT Center for Regulatory Science Harvard Medical School Boston Massachusetts USA

## Abstract

**Purpose:**

In the United States, the Food and Drug Administration has provided guidance on the contexts in which real‐world evidence (RWE) can be used to support regulatory decisions for medical devices.

**Methods:**

In this paper, we review different sources of real‐world data (RWD) for medical devices used in surgical procedures and how to optimize the design and analysis features of RWD studies to maximize the strength of RWE. The design and analysis of a study are critical to ensuring that the RWE generated from the RWD can ultimately be used to support a regulatory decision or fulfill a commitment for a medical device. We consider several topics relevant to medical device RWE in our review: misclassification and measurement error, causal inference, and incomplete follow‐up.

**Results:**

Some key challenges include indication and outcome misclassification in non‑registry data, tenuous assumptions of single‑arm designs, device‑ and surgeon‑level confounding in comparative studies, and potentially informative loss to follow‑up from censoring (e.g., insurance termination).

**Conclusion:**

The review is intended as a guide to researchers and regulators interested in generating or evaluating medical device RWE for regulatory purposes.

## Key Points

- Characteristics of RWD determine the sufficiency of the source data to generate RWE while the design and analysis of a study are critical to ensuring that the RWE can be used to support a regulatory decision.
- Clinical registries, non‐clinical registries, linked electronic health records, and hospital billing databases are among the most common sources of medical device RWD.
- For non‐registry data, indication and outcome misclassification represent important challenges to generating RWE.
- In medical device RWE studies the assumptions of single‐arm study designs are often tenuous because they unrealistically assume that the patient, surgeon, and hospital populations (and any other aspects of the study that influences the outcome) are the same in the target treatment and the data used to construct the performance goal, while comparative studies are challenged by confounding, particularly device‐ and surgeon‐level confounders.
- One source of incomplete follow‐up in RWE studies is discontinuing observation of all patients in a data source because a dataset is being prepared for analysis, in which case censoring is non‐informative and would not lead to bias, but other sources of censoring resulting in loss to follow‐up for individual‐specific reasons may be informative (e.g., health insurance termination) and can lead to bias, therefore sensitivity analyses are encouraged.

**Abbreviations**
CRN: coordinated registry network
EHR: electronic health record
FDA: Food and Drug Administration
IHDN: integrated healthcare delivery network
MDEpiNet: medical device epidemiology network
PPV: positive predictive value
RWD: real‐world data
RWE: real‐world evidence
UDI: unique device identifier
U.S.: United States
VISION: vascular implant surveillance and interventional outcomes
VQI: vascular quality initiative

## 1 Introduction

In the United States (US), the Food and Drug Administration (FDA) has described how it evaluates real‐world data (RWD) to determine whether it is sufficient for generating the types of real‐world evidence (RWE) that can be used in regulatory decisions for medical devices. Different types of RWD have different characteristics that determine whether the RWD can be used to support a particular regulatory decision, therefore it is essential that the accuracy of the data chosen in a particular application be sufficiently fit‐for‐use (e.g., premarket approval original applications, premarket notification 510 (k) submissions, post‐market surveillance studies, and post‐approval studies as a condition of approval [1, 2]). While the characteristics of an RWD source determine the sufficiency of the source data to generate RWE, the design and analysis of a study are critical to ensuring that the RWE can be used to support a regulatory decision.

FDA issued its final guidance in December of 2025 on the “Use of Real‐World Evidence to Support Regulatory Decision‐Making for Medical Devices” [1]. The document defines RWD as data relating to patient health status or the delivery of health care collected through electronic health records (EHRs), medical claims, product and disease registries, and other sources. To determine the suitability of RWD for a regulatory decision, FDA assesses the relevance and reliability of the data. Relevance includes the concepts of data availability, timeliness, and generalizability. Data availability refers to the RWD containing sufficient detail to capture the information needed to evaluate the question being addressed in the target population. Timeliness includes a sufficiently narrow time between data collection and release as well as the RWD reflecting the current clinical environment. Generalizability refers to the study sample being representative of the patients in the RWD source that are reflective of the proposed intended use population of the device. Reliability relates to data accrual, quality, and integrity. Accrual refers to data collected and processed in a consistent and methodical manner, including availability of information about data sources, accrual methods, and procedures. Quality and integrity encompass the presence of quality control processes; assessment of completeness, accuracy, and consistency across sites and over time; establishment and adherence to data collection, recording, and source verification procedures; adequate patient protections; and prior demonstration of RWE generation from the data source.

The purpose of this paper is as a methodological primer with two primary objectives: (1) to describe different sources of RWD for medical devices used in surgical procedures (e.g., including implantable devices but excluding wearables, external bracing devices) with consideration of strengths and limitations of each from a methodological perspective, and (2) to articulate key considerations related to study design and analysis from RWD sources, including: misclassification and measurement error, causal inference, and patient follow‐up. Recommendations on how to optimize use of RWD in light of each consideration is presented. Given the narrow scope of the review, non‐methodological topics are omitted and even certain methodological topics are not presented (e.g., sample size considerations).

## 2 Sources and Characteristics of RWD

Table 1 provides a sample of different RWD sources along with key characteristics. The most crucial element in any RWD source is device identification. Often device identification is based on manufacturer‐specific product codes, however FDA has developed and mandated use of Unique Device Identifiers (UDIs). UDIs follow internationally recognized standards and are registered in a centralized database (Global Unique Device Identification Database), and in turn enable more reliable device tracking, recall management, and regulatory oversight. Except for device registries, data collected as part of routine healthcare often does not contain information that can identify specific devices. The problem is that devices are not typically tracked in a way that can link to a patient's health record. Covariate information is important to the extent that it is used to identify the indication under study, characterize the sample, and adjust or balance for any differences in comparative studies. Registries are often limited to a pre‐determined set of covariates that are prospectively collected, while non‐registry sources are not limited in scope but instead by the granularity of information provided in clinical notes and codes. However, there are several examples of device registries linking to other data sources to obtain more complete data (e.g., outcomes not collected as part of the design of the registry or to increase length of follow‐up). This topic is described in greater detail in the paragraph on coordinated registry networks. The outcome collection methods, structure, and extent of follow‐up are key to determining the type of RWE that can be produced. For example, with implantable devices the most commonly assessed safety outcome is device replacement, and often this outcome is of interest over long periods of follow‐up.

**TABLE 1:** Types of RWD sources and associated characteristics.

Data source	Example	Device identification	Covariate information	Outcome ascertainment
Clinical Registry	Real‐World Atrial Fibrillation Registry	Yes	Yes, but possibly limited in scope to pre‐selected variables	Structured longitudinal follow‐up of outcomes typically evaluated in clinical studies. Good follow‐up in short‐ to medium‐term is possible (e.g., within 1 year of the index procedure).
Non‐Clinical Registry	Kaiser Permanente Total Joint Replacement Registry	Yes	Yes, but possibly limited in scope to pre‐selected variables	Unstructured longitudinal follow‐up of a more limited number of outcomes typically captured in clinical studies. Good follow‐up in short‐ to long‐term is possible.
Linked EHRs (e.g., IHDN)	Mercy Healthcare System	Possible	Yes, but requires extraction from clinical notes or codes	Unstructured longitudinal follow‐up of outcomes that rely on extraction from clinical notes or codes. Good follow‐up in short‐ to long‐term is possible
Hospital Billing Database	Premier Healthcare Database	Possible	Yes, but requires extraction from codes	Outcomes rely on extraction from codes. The extent of follow‐up is uncertain.

Clinical registries often retain many attractive features of a clinical study, such as device‐specific data, covariates relevant to the conditions of interest, and key outcomes carefully collected at pre‐specified timepoints. Consequently, the data elements are often regarded as having a high degree of accuracy, although missing data can be a problem, as well as limited covariates and length of follow‐up. One example of a clinical registry is the Real‐World Atrial Fibrillation Registry [3]. The registry, started in 2018, is a prospective observational multicenter registry of patients in the United States undergoing radiofrequency catheter ablation for symptomatic paroxysmal and persistent atrial fibrillation. Device product codes are recorded during the ablation procedure and detailed clinical information is collected prospectively prior to ablation, at the time of ablation, and at multiple times post‐ablation. This registry is well‐suited for regulatory purposes similar to those of clinical studies, such as for label expansion, but not for post‐approval and post‐marketing surveillance studies that require long‐term assessments beyond 12 months.

Non‐clinical registries target collection of data on a particular disease or device, but not with the degree of detail of a clinical registry. Commonly, physicians will contribute patient‐level data to a registry by completing a form describing the device, the indication being treated, and other summary‐level information regarding the patient and procedure. For example, the Kaiser Permanente Total Joint Replacement Registry [4] collects device‐specific data using product code labels, indication for arthroplasty, and other key information about the procedure through physician reporting. The registry is housed within Kaiser Permanente, an integrated health care delivery network (IHDN) and health insurer. Nesting a registry within an IHDN/health insurer allows the incorporation of data available through the health system and can support extended follow‐up of patients. This registry could be useful for post‐approval and post‐marketing surveillance studies that require assessments of a revision endpoint over long periods of time.

One common source of RWD is EHR data from an individual hospital. However, unless the hospital patient volume is quite large there will not be enough data to support a study for regulatory purposes, and there will be limited patient follow‐up (if patients seek care also in other hospitals) or the extent of follow‐up will be unknown. By comparison, hospitals that have linked EHRs, such as IHDN, where the healthcare provider has multiple facilities, can be useful to generate RWE because of its increased scale and patient follow‐up. While device identification is possible in a linked EHR, this relies on the network either being able to record use of specific devices at the point of care and linking that information to the patient EHR or the specific device being able to be extracted from the patient EHR (i.e., patient chart). Moreover, linked EHR are not specifically tailored to the therapeutic area of interest and therefore may be lacking data elements needed to support some regulatory applications. More generally, linked EHR rely upon codes that are populated as part of routine care to identify indications, confounders, and outcomes of interest. Patients are not followed up in a structured manner, rather each patient is followed‐up according to the standard of care provided by each physician, which can lead to delayed detection of safety events and make it challenging to evaluate effectiveness endpoints that are not routinely monitored by physicians. Mercy Health system is an example of an IHDN, consisting of 45 hospitals in the U.S. Midwest [5]. If patient‐level device data are available, as it is through device scanning in Mercy, IHDNs can be suitable for post‐approval and post‐marketing surveillance studies and may also have uses for label expansion [6]. Insurance claims databases are another source of RWD, but similar to many linked EHR will be lacking device‐specific data.

Another RWD source is a collection of unlinked hospital data in which the data of each hospital is aggregated into a single database. Outcomes over time are unlikely to be captured since longitudinality of the data are limited to patients repeatedly observed within the same hospital. One example is the Premier Healthcare Database [7], which aggregates billing data from more than 1400 hospitals throughout the United States as part of a group purchasing organization of hospitals. Device identification is possible through free text descriptions provided in the billing records.

Linkages among distinct types of data sources represent important advancements in generating RWD for medical devices by leveraging the strengths of multiple forms of data collection. Linkages of registries to other distinct data sources are also referred to as coordinated registry networks (CRN). Typically, CRNs combine an existing registry, which offer a high‐level of detail related to the device and procedure at baseline and outcomes in the short‐ to medium‐term, with a data source that can capture outcomes over a longer period through use of national or state‐based health data sources [8]. The vascular quality initiative (VQI) registry includes more than 550 participating centers in the United States, Canada, and Singapore, with detailed information on a variety of devices and procedures, though follow‐up is typically not past 1 year. One of the most mature examples of a CRN links the VQI registry to Medicare claims [9]. Generally, linkages among data sources create an opportunity to vastly improve the quality (e.g., increased follow‐up, addition of new outcomes and covariates) of any stand‐alone data source and are not limited to linkages with registries. Record linkages can improve data quality, but their use may be constrained by data‐use agreement, the cost of vendor‐provided linkage services, and delays inherent in maintaining linked datasets.

## 3 Select Considerations in Design and Analysis of Medical Device RWE Studies

There are numerous design and analysis considerations that arise in medical device RWE studies (e.g., [10]), but only a select few are considered in this paper. A summary of guidance on optimizing methods in light of these considerations is offered (Table 2).

**TABLE 2:** Summary of guidance for optimal methods in medical device RWE studies.

Key topic	Key subtopic	Guidance
Misclassification and measurement error	Indication identification	If using codes provide evidence supporting the validity of the phenotype algorithm (PPVs close to 1 preferred)
	Device identification	Use numerical device identifiers (e.g., product codes, UDIs) whenever available If device text descriptions are used consider using only very specific device descriptions
	Outcome ascertainment	Determine whether it is reasonable to assume non‐differential misclassification to better understand the potential direction of bias If using code‐based outcomes select a phenotype that maximizes specificity if using a ratio measure of effect, but if using an absolute measure of effect consider a phenotype that maximizes the sum of specificity and sensitivity. Consider the incidence of the outcome when determining whether a level specificity/sensitivity is adequate (may require simulation) If data are time‐to‐event consider whether measurement error in survival times will lead to earlier or later observed times and determine which direction this will bias the results The effect of any bias should be considered in light of the study objectives (e.g., the nature of the hypothesis test)
Causal inference	Single arm study	When comparing a study's result to a performance goal consider how patient, surgeon, and hospital factors in the studies used to construct the performance goal are different from the study in question and what bias may result
	Two‐arm comparative study	When multiple versions of a device exist and treatment effect heterogeneity is suspected, the treatment effect within each should be evaluated separately Address any device‐, surgeon‐, and hospital‐level confounding by covariate balancing Balance covariates without access to the outcome data Evaluate multiple balancing estimators for the same estimand Use high‐dimensional/large‐scale propensity scores when feasible Use negative outcome controls Use variance procedures that allow for non‐independence of observations within the same surgeon and/or hospital (e.g., cluster robust standard errors)
Incomplete follow‐up		End of follow‐up in RWD is unlikely to lead to a bias Loss‐to‐follow‐up can induce a bias and sensitivity analyses are encouraged (e.g., imputation of events/event times)

### 3.1 Misclassification and Measurement Error

Many RWD sources data elements can be subject to misclassification (discrete variables) and measurement error (continuous variables). Assessing the extent of misclassification is accomplished by comparing the method of data collection of interest (e.g., diagnosis codes) to some gold standard (e.g., charts) for assessing the same construct, and calculating certain operating characteristics (e.g., positive predictive value [PPV]) [11]. Misclassified treatment indication data pertains to uncertainty related to the indication under study. Misclassified indication data are less likely in registries and EHRs if the source is physician notes. Insurance claims and hospital billing data have a higher likelihood of misclassification as indications are often inferred from codes. For these data sources, evidence needs to be provided to support the phenotype algorithm that identifies the condition of interest. This may be from a past study that validates a set of codes, but ideally it would be from the same data source in which the study will be conducted, in order to limit concerns around transportability [12]. For indication, data we seek a PPV at or close to one. One problem that can arise is that the PPV is insufficiently high to identify an indication. One way to improve the PPV is to select codes that are more specific to the indication of interest, to require multiple specific codes (e.g., a combination of diagnosis, procedure, and prescription codes), and/or multiple specific codes over time [13].

A device can be identified based on its broad class (e.g., total knee replacement) or much more specifically with information that captures: the manufacturer, features that characterize an exact model, version of the model and lot identification. Although procedure codes can be used to identify a device class and are widely available in many RWD sources, in regulatory applications it is often the device‐specific information that is used in a study. While both a product code and UDI can capture a specific model, only UDI can identify versions of a model and specific lots, which can be important in certain applications. Product codes and UDIs can be entered either electronically (e.g., point‐of‐care barcode scanning) or manually. Although the latter may be prone to errors, if only select product codes/UDIs are considered for inclusion into a study, this will in most cases only have the effect of reducing the size of the eligible sample. Generally, device data in the form of a product code or UDI, often used in registries and select EHRs, is unlikely to lead to any misclassification. In contrast, some data sources may contain device data in less structured formats. For instance, the Premier Healthcare Database has device descriptions populated as free text. If descriptions that do not uniquely identify the device of interest are included in a study, this can lead to misclassification. This is problematic because the target treatment population is no longer correctly identified but also if the device has increased risk for a safety outcome (and the misclassified devices do not) then this can lead to attenuation of the treatment effect. Typically, there are fewer opportunities to validate device text descriptions because a gold standard is unavailable, therefore one strategy is including only data on patients with very detailed device descriptions. However, if physician‐populated data are being used to identify devices, it is possible that this strategy can introduce a (selection) bias, if the level of detail provided by physicians is related to the outcome (e.g., physicians providing less detailed information on devices also have worse outcomes).

Misclassification and measurement errors in outcome data lead to problems related to accuracy of estimates. Outcome misclassification is not generally a problem in registry data, but is in other data sources where identification of the outcome relies on code‐based phenotypes. In comparative studies, the bias depends on the degree of the misclassification, the estimator, and outcome incidence [11, 14]. Assuming no other sources of bias, a risk ratio can produce an unbiased estimate of effect even when the outcome measure has low sensitivity, as long as the level of sensitivity is non‐differential between exposure groups and specificity is 100% [11]. In practice, investigators often select outcome definitions that are expected to have high specificity under the assumption that near‐perfect specificity will lead to risk ratio estimates that are close to unbiased, but even very high specificities can still lead to considerable downward bias when the outcome is rare [15]. For the risk difference, the degree of bias is linearly related to the sum of the decreases in sensitivity and specificity and is independent of the outcome incidence [14]. The same pattern generally holds for time‐to‐event data except the downward bias is magnified when measurement errors lead to earlier survival times [15]. The consequence is a bias toward the alternative hypothesis in a comparative non‐inferiority labeling expansion study and bias toward the null hypothesis in superiority post‐market safety studies. Ultimately, the onus is on the researcher to demonstrate that an outcome phenotype will not result in considerable bias in the study population of interest, and if it does, that it is conservative with respect to the intended objective and that the design can accommodate the expected downward bias.

### 3.2 Causal Inference

For the types of regulatory studies considered in this paper a common goal is to quantify the strength of a causal relationship. All studies are subject to some degree of bias (and sampling error); therefore, the extent to which we can accurately answer a causal question from a study is a matter of degree. While the assumptions and methods for causal inference may be more familiar in a comparative study, the same goal also applies in single‐arm studies, which are commonplace in medical device research.

Many medical device clinical studies used for regulatory approval of new devices use a single‐arm study design. Similarly, single‐arm RWE studies are also used quite often [2]. Whenever single‐arm studies are used, patients receiving the device of interest are compared to a fixed quantity, a performance goal. A performance goal is the maximum tolerated value for a safety endpoint or minimum tolerated value for an efficacy endpoint. The performance goal approach compares a device of interest to a standard of care and determines whether the device of interest causes outcomes that are substantively similar, less harmful, or more beneficial. Key to drawing one of these study conclusions is establishing that the research contexts are identical or that there are no cross‐study differences. Strictly speaking this implies that the patient, surgeon, and hospital populations involved in patient treatment are the same, as well as any other aspects of the study that influence the outcome (e.g., measurement accuracy of the outcome). These assumptions are often tenuous and only possible to evaluate for measured characteristics.

A two‐arm comparative design is often used with RWD but also carries with it certain assumptions for causal inference, including: no unmeasured confounding (exchangeability), positivity, no interference, and stable treatment [16]. Stable treatment means that each patient receives the same version of treatment, or if multiple versions of a treatment do exist, then they have the same effect on the outcome [16]. When treatment refers to the use or implantation of a device, often there are multiple versions of a device (e.g., based on size or material) that are grouped into a single treatment group. When this is the case and treatment effect heterogeneity is suspected, one option is partitioning the treatment group into more homogeneous device subgroups and evaluating the treatment effect within each.

The assumption of no unmeasured confounding is also necessary for making causal inferences. Typically, only pre‐treatment patient attributes are considered as potential confounders, but device‐level confounders are also possible and, if not addressed, would lead to unmeasured confounding. Device‐level confounding are devices used concurrently with the device of interest, common to both the treated and comparison groups, and associated with the outcome. For example, in hip replacements, the four major components are a femoral stem, a femoral head, an acetabular shell, and an acetabular liner. For each of these four components, there are numerous variations based on design, size, and material. If interest is in the treatment effect of one component, then an attempt to measure and balance on other component characteristics should be made. The safety and effectiveness of the device can also be influenced by the skill of the surgeon, and surgeons preferentially select certain devices over others; therefore, there can be confounding effects related to the surgeon [10, 17]. There may also be hospital‐level variables that act as confounders.

Regardless of the source of confounding, adjustment is needed to mitigate bias. Covariate balancing methods based on the propensity score are often advocated in regulatory applications [18] and the context in which we consider analytical solutions presented below. Among the best practices include: balancing of covariates without access to the outcome data to limit the potential for investigator bias [18], considering multiple balancing estimators for the same estimand and choosing the method that maximizes balances and (secondarily) effective sample size [19, 20], addressing unmeasured confounding via the use of high‐dimensional or large‐scale propensity scores when feasible [21, 22] and use of negative outcome controls [23]. In the context of covariate balancing, the extent of the surgeon experience with a device is often quantified by covariates representing number of uses of the device or average volume over some fixed period of time. When a device is new to the market, analyses may be limited to observations after a surgeon has sufficient experience with the device (i.e., excluded time is known as a run‐in period). These approaches may not adequately capture all aspects of a surgeon's experience however, therefore other approaches that can be used to balance on unmeasured time‐invariant surgeon‐level factors include: within surgeon matching, fixed/random surgeon effects and preferential within‐surgeon matching [24], and these can be generalized to account for time‐dependent effects [25]. Finally, a somewhat separate issue is non‐independence of observations due to patients within the same surgeons and hospitals having similar outcomes. Here the concern is not bias of estimates, but rather underestimation of variance leading to confidence intervals that have less than nominal coverage and liberal hypothesis tests. Two solutions to address this problem are cluster robust standard errors and bootstrapping [17].

### 3.3 Incomplete Follow‐Up

The extent and reasons for incomplete patient follow‐up can have important consequences on the quality of RWD that is collected. In clinical studies of medical devices, the reason for incomplete follow‐up may be related to diminished safety or effectiveness of the device of interest and could lead to bias in its evaluation. A common approach for binary endpoints in single‐arm medical device clinical studies is to perform a tipping point sensitivity analysis [26]. In contrast to clinical studies, in most RWE studies (not including clinical registries), one cause of incomplete follow‐up is end of follow‐up in the data source. In survival analysis, the methods by which most RWE are analyzed, discontinuing observation of all patients in a data source because a dataset is being prepared for analysis is non‐informative and would thus not induce a bias [27]. By contrast, when patients are lost to follow‐up for individual‐specific reasons (e.g., healthcare membership termination in insurance‐based databases), this could be an informative cause of censoring in RWD if the loss to follow‐up is due to a systematic worsening or improvement in health status, which would lead to a bias. Unfortunately, it is not possible to determine empirically whether censoring is informative or non‐informative, but sensitivity analyses are encouraged. One option is a worst‐case/best‐case approach, where in the worst‐case approach patients who are censored due to a particular cause are assumed to experience the outcome immediately following their being censored [28]. Another method imputes the outcome (event and event times) such that censored observations are assumed to have increased or decreased risk of the event of interest [29].

## 4 Conclusions

In recent years, the FDA has described the contexts in which RWE can be used to support regulatory decisions [1, 2]. In this paper, we have described different sources of RWD and how to optimize design and analysis features to maximize the strength of evidence generated using these data sources. While an RWD source may be adequate to achieve its regulatory objective, without a rigorous study design and analysis, the resulting evidence may not be adequate to support a regulatory decision.

The intent of this review was to draw attention to data sources and methods most relevant to medical device research. Given the scope of the review, we do not cover all types of RWD sources or all methods that are needed for generating RWE. This review has highlighted key considerations in selecting data sources and study methods when generating medical device RWE for regulatory purposes. Collectively, the use of high‐quality RWE has the potential to produce timely, cost‐efficient, and generalizable evidence that can complement prospective clinical studies.

## Funding

The authors have nothing to report.

## Conflicts of Interest

Guy Cafri and Paul Coplan are employees of Johnson & Johnson.

## Data Availability

Data sharing not applicable to this article as no datasets were generated or analysed during the current study.
